# Carbon Nanotube-Based Field-Effect Transistor Biosensors for Biomedical Applications: Decadal Developments and Advancements (2016–2025)

**DOI:** 10.3390/bios15050296

**Published:** 2025-05-07

**Authors:** Joydip Sengupta, Chaudhery Mustansar Hussain

**Affiliations:** 1 Department of Electronic Science, Jogesh Chandra Chaudhuri College, Kolkata 700033, India; joydipdhruba@gmail.com; 2 Department of Chemistry and Environmental Science, New Jersey Institute of Technology, Newark, NJ 07102, USA

**Keywords:** CNT-FET biosensors, functionalization strategies, biomarker detection, device architectures, commercialization

## Abstract

Advancements in carbon nanotube-based FET (CNT-FET) biosensors from 2016 to 2025 have boosted their sensitivity, specificity, and rapid detection performance for biomedical purposes. This review highlights key innovations in transducer materials, functionalization strategies, and device architectures, including floating-gate CNT-FETs for detecting cancer biomarkers, infectious disease antigens, and neurodegenerative disease markers. Novel approaches, such as dual-microfluidic field-effect biosensor (dual-MFB) structures and carboxylated graphene quantum dot (cGQD) coupling, have further expanded their diagnostic potential. Despite significant progress, challenges in scalability, reproducibility, and long-term stability remain. Overall, this work highlights the transformative potential of CNT-FET biosensors while outlining a roadmap for translating laboratory innovations into practical, high-impact biomedical applications.

## 1. Introduction

Biosensors play a crucial role in modern diagnostics in the rapid, sensitive, and specific detection of biomolecules for biomedical applications [1]. Among various biosensing platforms, CNT-FET biosensors have emerged as a highly promising class of devices due to their exceptional electrical properties, high surface area, and ability to be functionalized for selective biomolecular interactions [2]. Over the past few years, advancements in CNT-FET biosensor technology have led to significant improvements in sensitivity, detection limits, and real-time monitoring capabilities, making them valuable tools for disease diagnosis and bioanalytical applications [3].

The unique properties of CNTs, including their high carrier mobility, excellent conductivity, and nanoscale dimensions, allow for enhanced signal transduction when integrated into FET-based biosensors [4]. Decadal developments have focused on optimizing transducer materials, refining functionalization strategies to improve biomolecular selectivity, and innovating device architectures to enhance overall performance. Notably, novel designs such as floating-gate CNT-FETs [5], dual-MFB structures [6], and cGQD-coupled configurations [7] have expanded the capabilities of these sensors in detecting cancer biomarkers, infectious disease antigens, and neurodegenerative disease markers with unprecedented precision.

Despite these remarkable advancements, several challenges hinder the widespread commercialization and practical deployment of CNT-FET biosensors [8]. Issues related to scalability, reproducibility, and long-term operational stability must be addressed to ensure consistent performance across diverse biomedical applications [9]. Additionally, integrating these sensors into portable, cost-effective diagnostic platforms remains a key focus for future research and development [10].

**Scheme 1:**
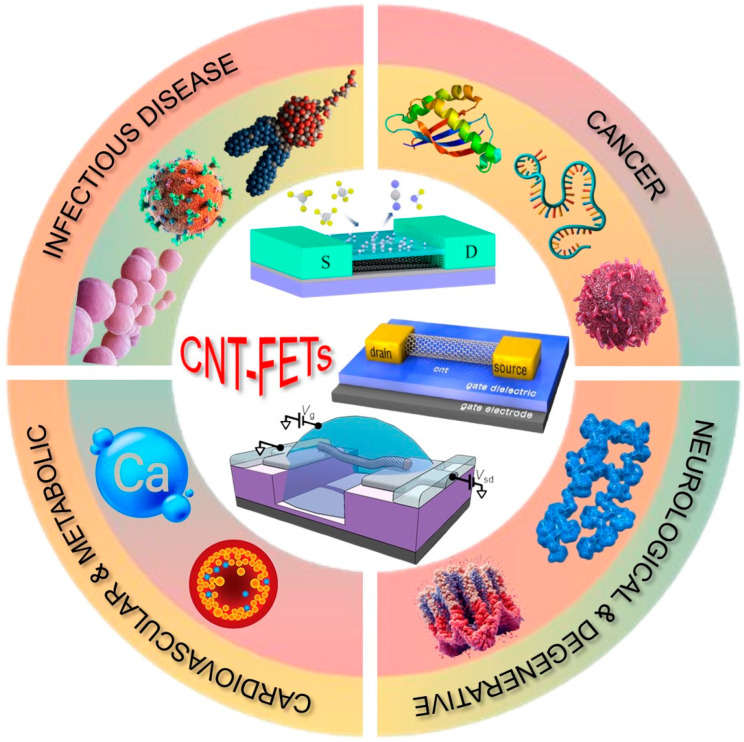
Overview of CNT-FET-based biosensors for detecting biomarkers across major disease categories, including infectious, cancerous, neurological, and cardiovascular/metabolic disorders.

This review provides an overview of decadal progress in CNT-FET biosensors from 2016 to 2025, highlighting innovative transducer materials, functionalization techniques, and novel device architectures. Furthermore, it discusses the existing challenges and potential solutions for translating these laboratory-based advancements into commercially viable biosensing platforms. By exploring the latest trends and emerging strategies, this review aims to contribute to the ongoing efforts to harness CNT-FET biosensors for high-impact applications in healthcare and beyond (Scheme 1).

## 2. Carbon Nanotubes: Types, Synthesis, and Properties

**Figure 1:**
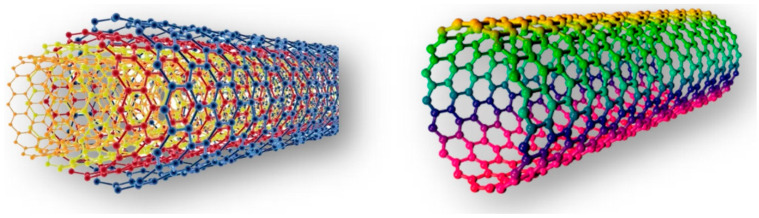
The nanostructure of a multi-wall carbon nanotube (MWCNT) (**left**) and a single-wall carbon nanotube (SWCNT) (**right**) (adapted with permission from Ref [17]).

Carbon nanotubes (CNTs) are one of the most extensively studied nanomaterials due to their exceptional electrical, mechanical, and thermal properties [11]. Structurally, they are composed of rolled-up graphene sheets, forming hollow cylindrical tubes with nanometer-scale diameters and micrometer-scale lengths. Depending on their structure, CNTs can be classified as single-walled CNTs (SWCNTs) or multi-walled CNTs (MWCNTs) (Figure 1). SWCNTs consist of a single graphene layer and exhibit unique electronic properties governed by their chirality, allowing them to behave as either metallic or semiconducting materials. In contrast, MWCNTs comprise multiple concentric graphene cylinders and generally possess superior mechanical strength with metallic conductivity [12]. Due to their remarkable properties, CNTs have found extensive applications in fields such as nanoelectronics [13], biosensors [14], composite materials [15], and energy storage [16].

The synthesis of CNTs plays a crucial role in determining their structural quality, yield, and applicability. Several methods have been developed for their fabrication, with the most common techniques being arc discharge [18], laser ablation [19], and chemical vapor deposition (CVD) [20]. Arc discharge and laser ablation methods rely on the high-temperature vaporization of graphite, producing high-purity CNTs but with limitations in scalability and cost. In contrast, CVD has emerged as the most widely used technique due to its ability to control CNT diameter, length, and alignment through catalyst-assisted hydrocarbon decomposition at moderate temperatures. A variation of this process, plasma-enhanced chemical vapor deposition (PECVD) [21], enables the fabrication of vertically aligned CNTs [22], which are particularly useful for nanoelectronic and sensing applications [23].

The unique properties of CNTs make them highly attractive for advanced technological applications. Their electronic characteristics [24], dictated by chirality and quantum effects [25,26], enable ballistic electron transport [27] with an extremely high level of carrier mobility, making them ideal for nanoelectronic devices such as FETs and interconnects. Mechanically, CNTs possess extraordinary levels of tensile strength and flexibility, with a huge Young’s modulus [28]. Their superior thermal conductivity makes them excellent candidates for heat dissipation in electronic devices [29]. Additionally, CNTs exhibit strong optical absorption in the near-infrared region, enabling applications in the biomedical sector [30].

CNT detection technology has emerged as a transformative approach in various sensing applications due to its unique physicochemical properties [31]. One of the primary advantages of CNTs is their exceptional electrical conductivity, which enables the highly sensitive detection of analytes at low concentrations. The high surface-to-volume ratio of CNTs facilitates enhanced interaction with target molecules, improving the signal-to-noise ratio in sensors. Additionally, CNTs exhibit remarkable mechanical strength and chemical stability, ensuring durability and reliability in harsh environments. Their versatility allows functionalization with diverse chemical groups, enabling tailored specificity for detecting biomolecules. Furthermore, CNT-based sensors are compact and energy-efficient, making them suitable for integration into portable devices [32].

## 3. CNT-FET Biosensors

### 3.1. Basic Configuration and Advanced Architectures

**Figure 2:**
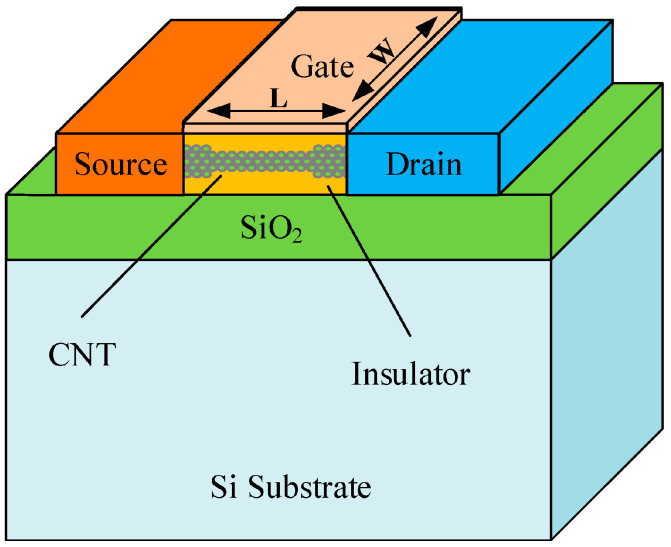
Device structure of a CNT-FET (reproduced with permission from [36]).

CNT-FET biosensors have emerged as a highly promising platform for ultra-sensitive and selective biomolecular detection. These biosensors utilize CNTs as the active channel material, offering exceptional electronic properties, including a high level of carrier mobility and low noise levels. The basic configuration of a CNT-FET biosensor [33] consists of three main components: the source and drain electrodes, the semiconducting CNT channel, and a gate terminal that modulates the device’s electrical properties (Figure 2). The target biomolecule interacts with functionalized CNTs [34], inducing changes in the local electrostatic environment, which alters the transistor’s conductivity. This real-time, label-free detection mechanism makes CNT-FET biosensors highly effective for applications in medical diagnostics and point-of-care testing [35].

While the fundamental CNT-FET biosensor configuration provides significant advantages in sensitivity and miniaturization, advanced architectures have been developed to further enhance their performance, specificity, and integration capabilities. Floating-gate CNT-FETs incorporate an additional gate electrode, enabling memory-like sensing functions and improved signal stability [37]. Liquid-gated CNT-FETs, in which the electrolyte solution serves as the gate medium, enhance biocompatibility and allow for direct interactions between biomolecules and the transistor channel [38]. Another innovative structure is the dual-gated CNT-FET [39], which introduces an additional control gate to modulate the charge distribution in the channel, thereby improving the detection sensitivity and reducing noise. Additionally, multi-fingered back-gated CNT-FET designs enhance signal amplification, making them suitable for detecting ultra-low concentrations of analytes [40].

**Figure 3:**
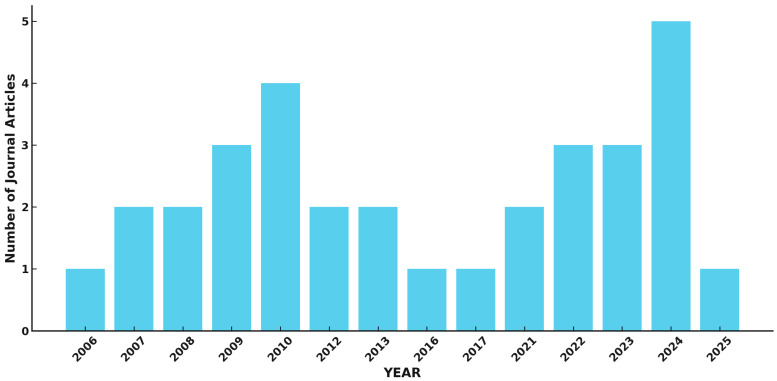
Research trends for CNT-FET biosensors based on data retrieved from Scopus (Elsevier) using the following search query: TITLE (cnt OR ‘carbon nanotube’ AND fet OR ‘field effect transistor’ AND biosensor).

Further advancements in CNT-FET biosensors involve nanomaterial integration to optimize detection capabilities. cGQD-coupled CNTs have demonstrated enhanced sensitivity and selectivity, particularly for bacterial toxin detection [7]. Additionally, dual-MFB structures improve the level of specificity for small-molecule detection by mimicking natural binding sites [6]. The use of semiconducting SWCNTs enables label-free detection in complex biological matrices, significantly improving real-time biosensing applications. Hybrid architectures integrating other nanomaterials, such as graphene [41] and metal nanoparticles [42], further optimize the charge transfer efficiency and enhance biosensor performance. Moreover, flexible and stretchable CNT-FET biosensors are being explored for wearable and implantable biomedical applications, offering real-time health monitoring capabilities [43]. Figure 3 illustrates the research progression and emerging trends in CNT-FET biosensor development.

### 3.2. Functionalization Strategies for Enhanced Biosensing

Functionalization strategies [44] play a crucial role in enhancing the performance of CNT-FET biosensors by improving selectivity, stability, and sensitivity. The incorporation of bio-recognition elements onto CNT surfaces [45] enables the highly specific detection of target biomolecules. Aptamer-functionalized CNT-FETs have demonstrated remarkable potential for single-pathogen detection, such as identifying Salmonella enterica with high precision [46]. Similarly, antibody-conjugated CNT biosensors facilitate the detection of disease-specific biomarkers, including the SARS-CoV-2 spike protein [47], enabling rapid and accurate diagnostics. Additionally, DNA hybridization probes have been employed for microRNA (miRNA) and genomic analysis [48], offering a powerful platform for genetic screening and early disease detection. These bio-functionalization approaches ensure that CNT-FET biosensors can achieve molecular-level specificity, making them highly effective for biomedical applications.

Beyond bio-recognition elements, surface modification techniques further enhance CNT-FET biosensor performance by improving biomolecule immobilization and optimizing electrical properties. PBASE (1-pyrenebutyric acid N-hydroxysuccinimide ester) linker chemistry [47] has been widely employed for the stable and efficient attachment of biomolecules onto CNT surfaces, ensuring long-term sensor functionality. Polymer doping strategies, such as the use of polyethyleneimine (PEI) and polypyrrole, [49] have been explored to modulate CNT conductivity and enhance overall sensing performance. These polymer-based modifications can significantly improve the charge transfer efficiency and signal transduction in biosensing applications.

In addition to polymer-based enhancements, nanomaterial decoration techniques have been implemented to amplify signal responses in CNT-FET biosensors. The integration of metal nanoparticles, such as gold (Au-NPs) [48], onto CNT surfaces facilitates superior electron transport and enhances biomolecular interactions, leading to improved detection sensitivity. These nanoparticles provide localized surface plasmon resonance (LSPR) effects [50], further boosting signal amplification. The combination of bio-recognition elements, stable surface modifications, and nanoparticle-based signal enhancement strategies contributes to the development of next-generation CNT-FET biosensors with unparalleled sensitivity, selectivity, and real-time detection capabilities. These advancements pave the way for their integration into clinical diagnostics and personalized healthcare applications.

## 4. Applications of CNT-FET Biosensors in Disease Diagnostics

### 4.1. Cancer Detection

Early cancer detection is critical for improving survival rates, yet traditional methods like biopsies and imaging are often invasive and costly. CNT-FET biosensors enable the rapid, label-free detection of biomarkers such as miRNA or exosomal protein for breast cancer and CEA for lung cancer, allowing for early diagnosis and personalized treatment. Their high sensitivity and real-time capabilities make them a promising alternative to conventional diagnostic tools.

**Figure 4:**
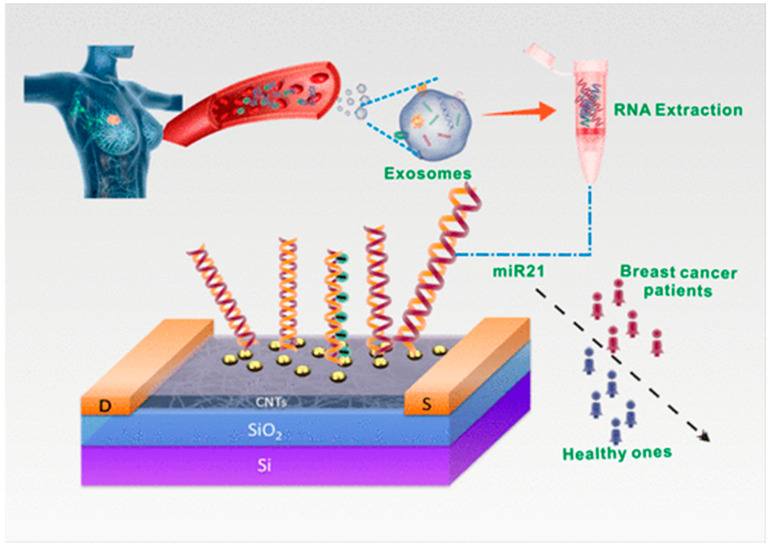
Graphical representation of the ultrasensitive detection of exosomal miR-21 employing a DNA-functionalized CNT-FET biosensor (reproduced with permission from [48]).

Li et al. [48] demonstrated that tumor-derived exosomal miRNAs play critical roles in cancer initiation and progression, representing promising candidates for early disease detection and therapeutic monitoring. They addressed the persistent challenge of achieving the simple, label-free, and highly sensitive detection of these biomarkers through the development of an advanced FET biosensing platform (Figure 4). This innovative device utilized a polymer-purified semiconducting CNT film with exceptional electronic properties, departing from conventional designs through the implementation of a floating-gate architecture. The investigators employed an yttrium oxide dielectric layer of minimal thickness, subsequently decorating the surface with gold nanoparticles to serve as molecular anchoring sites. A thiol-modified DNA probe was covalently attached to the nanoparticle-modified sensing interface, enabling the specific capture of target miRNA through hybridization events detectable via electrical signal modulation. The biosensing platform exhibited remarkable analytical performance, combining an exceptional level of detection sensitivity with stringent molecular discrimination capabilities. Clinical validation studies utilizing human plasma specimens revealed statistically significant differentiation between healthy controls and breast cancer patients, confirming the diagnostic potential of this nanotechnology-based approach. These findings established the developed CNT-FET biosensor as a viable tool for non-invasive cancer diagnostics through exosomal miRNA profiling.

Exosomal proteins serve as clinically relevant biomarkers for numerous pathological conditions, creating significant demand for highly sensitive detection methodologies. The research team of Li et al. [51] developed an innovative FET biosensing platform utilizing polymer-purified semiconducting CNT thin films for the label-free identification of MUC1, a transmembrane glycoprotein overexpressed in breast-cancer-derived exosomes. While polymer-sorted semiconducting nanotubes offered exceptional electronic properties and processing efficiency, their chemical inertness presented challenges for biomolecular functionalization. To address this limitation, the investigators implemented a poly-L-lysine surface modification strategy following nanotube deposition within the transistor channel region. The functionalized platform incorporated gold nanoparticles as scaffolding elements for thiolated aptamer probe immobilization, enabling the specific capture of target exosomal proteins. The optimized biosensor demonstrated outstanding analytical performance in discriminating MUC1-positive exosomes with remarkable levels of sensitivity and molecular specificity. Clinical validation experiments successfully differentiated plasma samples from breast cancer patients and healthy donors based on exosomal MUC1 expression profiles.

Examinations of the BRCA1 gene emerged as a critical strategy for liquid biopsy and early breast carcinoma identification, yet its broad implementation was constrained by the elevated costs, extended processing times, and intricate nature of traditional sequencing methodologies. A novel DNA sensor, leveraging CNTs within an FET framework, incorporated a floating-gate architecture and a high-κ dielectric bilayer of Y_2_O_3_ and HfO_2_. A pivotal innovation, as reported by Liu et al. [52], involved the systematic assessment of three nucleic acid probes (deoxyribonucleic acid, phosphorodiamidate morpholino oligomers, and peptide nucleic acid) to ascertain the most effective configuration for BRCA1 detection. The peptide-nucleic-acid-modified sensor exhibited an exceptional analytical sensitivity, achieving a limit of detection of 1.38 aM, alongside a robust specificity. Additionally, the sensor demonstrated significant resilience against interference in serum-based BRCA1 assays, attributed to the protective properties of the dielectric bilayer. Clinical evaluations confirmed the sensor’s capacity to accurately differentiate breast cancer patients from healthy controls, yielding results congruent with established sequencing techniques. This investigation established a streamlined, highly sensitive, and precise diagnostic platform for breast cancer liquid biopsy, offering valuable guidance for the development and optimization of subsequent DNA biosensing technologies, particularly in the selection of molecular recognition components.

**Figure 5:**
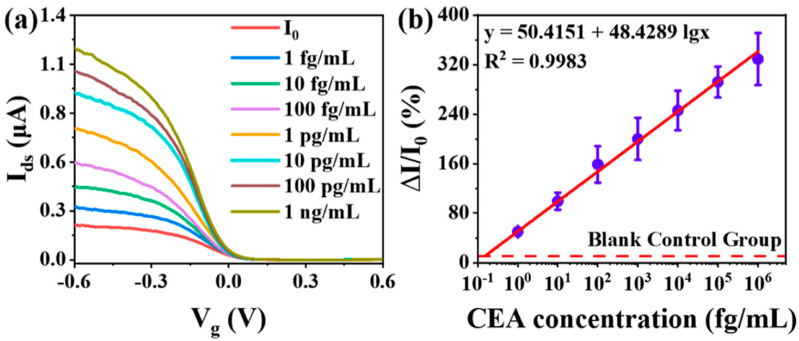
Detection of CEA using the proposed CNT-FET biosensor with an undulating interface in a complex matrix: (**a**) The transfer characteristics of the CNT-FET biosensor were systematically monitored in a 10% fetal bovine serum (FBS) matrix following the addition of CEA at concentrations ranging from 1 fg/mL to 1 ng/mL, demonstrating the sensor’s performance in biologically relevant conditions. (**b**) Corresponding calibration curves illustrating the relative current change (ΔI/I_0_) as a function of increasing CEA concentration were generated, with error bars representing the standard deviation across eight independently fabricated devices (n = 8), underscoring measurement reproducibility and device reliability (reproduced with permission from [5]).

Li et al. [5] identified carcinoembryonic antigen as an established biomarker for lung carcinoma detection, though its full clinical potential remained unrealized due to limitations in current analytical methodologies regarding sensitivity and dynamic range. The researchers developed an advanced floating-gate FET biosensing platform incorporating semiconducting CNT thin films paired with a nanostructured yttrium oxide dielectric layer to address these diagnostic challenges. This innovative architecture employed a deliberately engineered undulating biosensing interface that simultaneously enhanced the probe density and interfacial capacitance, thereby improving both the detection range and sensitivity beyond those of conventional transistor-based sensors. Systematic characterization revealed the nanostructured dielectric surface provided optimal conditions for biorecognition element immobilization while significantly boosting biosensor performance parameters. The platform demonstrated robust operation in complex biological matrices, maintaining analytical reliability when challenged with fetal bovine serum samples (Figure 5). These findings suggested the developed CNT-FET configuration possessed substantial potential for clinical implementation in early-stage lung cancer diagnostics through carcinoembryonic antigen detection.

### 4.2. Infectious Disease Diagnosis

The rapid and accurate detection of infectious diseases is essential for controlling outbreaks and ensuring timely treatment. Traditional methods like PCR are effective but require specialized labs and long processing times. CNT-FET biosensors offer the portable, ultra-sensitive detection of pathogens, such as SARS-CoV-2 for COVID-19 and Salmonella or Staphylococcus for foodborne diseases. Their ability to provide real-time results makes them ideal for point-of-care diagnostics and epidemic management.

**Figure 6:**
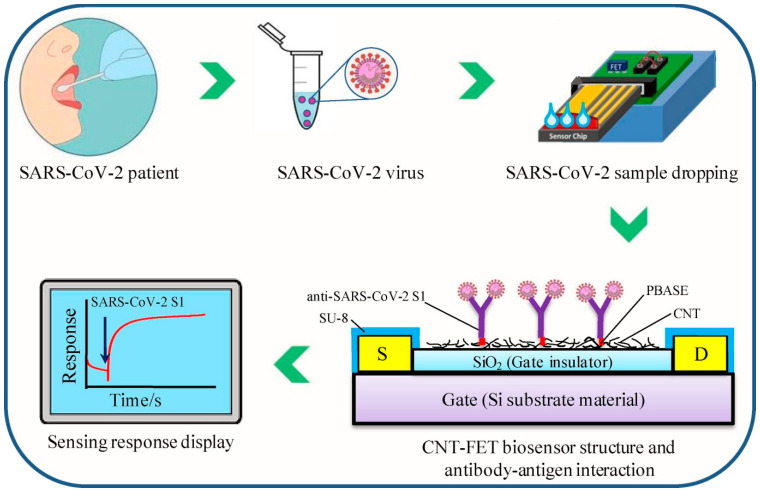
A schematic representation of the CNT-FET biosensor and the testing procedure for SARS-CoV-2 S1. The sensor utilizes SWCNT as the sensing nanomaterial, with anti-SARS-CoV-2 S1 immobilized on the CNT via the PBASE linker. The detection mechanism relies on changes in electrical signal properties induced by the interaction between the biosensor and the SARS-CoV-2 spike protein (reproduced with permission from [47]).

Zamzami et al. [47] recognized that widespread diagnostic testing for SARS-CoV-2 infection represented a critical need during pandemic situations for effective disease surveillance and clinical management. They developed a rapid, cost-effective electrochemical biosensing platform employing CNT-FET that was capable of digitally quantifying viral antigens in saliva specimens (Figure 6). The device fabrication involved the precise deposition of CNTs onto silicon substrates followed by functionalization with specific anti-SARS-CoV-2 S1 antibodies through pyrene-based linker chemistry. A systematic evaluation demonstrated the biosensor’s capacity to selectively identify target viral antigens while exhibiting no cross-reactivity with structurally related coronavirus proteins. Analytical characterization revealed an exceptional detection sensitivity across a broad dynamic range in optimized buffer conditions. The platform’s performance in complex biological matrices suggested its potential utility in point-of-care applications, offering significant advantages in testing throughput and operational simplicity compared to conventional diagnostic methodologies. These findings established the developed CNT-FET platform as a promising solution for mass screening applications during public health emergencies requiring rapid viral detection capabilities.

Cui et al. [7] identified blood endotoxin as a critical pathogenic factor capable of inducing severe febrile conditions and life-threatening endotoxemia, necessitating the development of rapid and sensitive detection methodologies. They fabricated a CNT-FET biosensing platform for label-free endotoxin monitoring, employing polymer-purified semiconducting nanotube films as the conductive channel. To enhance the analytical sensitivity, cGQDs were integrated onto the nanotube surface through poly-L-lysine-mediated conjugation, serving as nanoscale scaffolds for the subsequent immobilization of polymyxin B recognition elements. This molecular architecture demonstrated an exceptional binding specificity toward endotoxin molecules while maintaining robust performance in complex biological matrices. A systematic evaluation revealed the biosensor’s capacity to identify Gram-negative bacterial infections in clinical blood specimens with remarkable diagnostic accuracy, as validated through an analysis of receiver operating characteristics. The platform’s combination of rapid response characteristics, interference resistance, and clinically relevant detection thresholds established its potential as an early warning system for sepsis-related conditions.

Feng et al. [46] recognized that foodborne pathogens including Salmonella enterica and Staphylococcus aureus represent substantial public health threats, creating an urgent need for advanced detection platforms capable of rapid and accurate pathogen identification. The researchers developed a CNT-FET biosensing system incorporating nucleic acid aptamers specifically engineered for high-affinity microbial capture. This platform demonstrated exceptional analytical performance, achieving pathogen detection at the single-cell level while maintaining rigorous specificity standards. A systematic evaluation revealed the biosensor’s capacity to maintain its detection efficacy in complex food matrices, overcoming interference challenges commonly encountered in real-world applications. The platform exhibited rapid response characteristics without compromising sensitivity, representing a significant advancement over conventional microbiological detection methods. These findings suggest the aptamer-functionalized transistor architecture holds substantial potential for deployment in food safety monitoring applications requiring portable, label-free detection capabilities. The investigation established that nanomaterial-enhanced biosensing technologies could effectively address critical gaps in current pathogen surveillance methodologies while meeting the demanding requirements of field-deployable diagnostic systems.

### 4.3. Neurological and Degenerative Disease Monitoring

Neurodegenerative diseases like Alzheimer’s and osteoarthritis often go undetected until reaching advanced stages. Current diagnostic techniques rely on costly imaging or invasive procedures. CNT-FET biosensors enable the early, non-invasive detection of biomarkers like amyloid-beta for Alzheimer’s and inflammatory markers for osteoarthritis. Their rapid, high-precision capabilities improve early diagnosis and disease management.

Marques et al. [53] developed CNT-based disposable FET biosensors (bio-FETs) to detect domoic acid (DA), a biotoxin linked to amnesic shellfish poisoning. The bio-FET’s analytical performance was benchmarked against an enzyme-linked immunosorbent assay (ELISA), a conventional method, to confirm its efficacy for DA detection. Calibration curves were established using standard DA solutions ranging from 10 to 500 ng/L, with five bio-FETs employed to assess reproducibility and dual measurements per sensor to evaluate repeatability. They validated the bio-FET using ten artificially spiked seawater samples. The sensors demonstrated a reproducibility of 0.52–1.43%, a repeatability of 0.57–1.27%, a limit of detection of 10 ng/L, and a recovery range of 92.3–100.3%, indicating robust analytical capability for DA quantification in environmental seawater samples.

**Figure 7:**
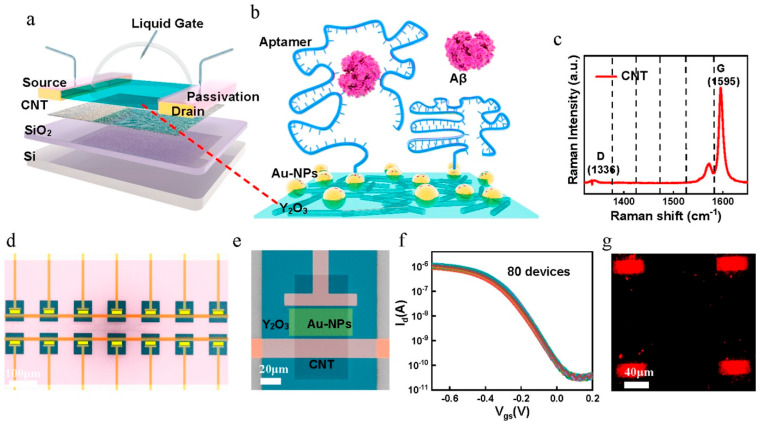
Development and analysis of the CNT-FET biosensor. (**a**) Conceptual representation of an aptamer-modified CNT-FET biosensor designed for detecting AD serum biomarkers. (**b**) Schematic depiction of the immobilization process, wherein an aptamer probe is anchored onto FG via a conventional Au linker, subsequently enabling hybridization with Aβ peptides. (**c**) Raman spectroscopy image illustrating the distribution of sorted CNTs. (**d**) Scanning electron microscopy (SEM) image displaying the CNT-FET biosensor array, with a scale bar of 100 μm. (**e**) SEM image highlighting the core active region of the biosensor, with a scale bar of 20 μm. (**f**) Transfer characteristics of 80 CNT-FETs under liquid-gated conditions following the deposition of Au nanoparticles, measured at V_ds_ = −0.1 V. (**g**) Fluorescence microscopy image revealing Cy5-labeled Aβ_42_ aptamers hybridized to Au-NPs immobilized on the CNT-FG-FET channel surface (reproduced with permission from [54]).

Chen et al. [54] reported the critical role of blood-based biomarkers in early Alzheimer’s disease diagnosis while recognizing the analytical challenges posed by the complex serum matrix and ultra-low biomarker concentrations. They addressed these limitations through development of a scalable CNT-FET platform incorporating uniform semiconducting CNT thin films for β-amyloid peptide detection (Figure 7). This mass-producible biosensor architecture, when combined with high-affinity oligonucleotide aptamers, demonstrated exceptional analytical performance in undiluted human serum, surpassing existing detection methodologies in both sensitivity and reproducibility. Through the systematic optimization of surface blocking protocols, the platform achieved remarkable molecular discrimination capabilities while minimizing non-specific adsorption effects. The biosensor exhibited wide dynamic range characteristics coupled with rapid response kinetics and minimal operational variability, establishing its potential as a cost-effective diagnostic solution. These advancements represent significant progress toward translating complex laboratory assays into practical point-of-care applications for neurodegenerative disease screening.

**Figure 8:**
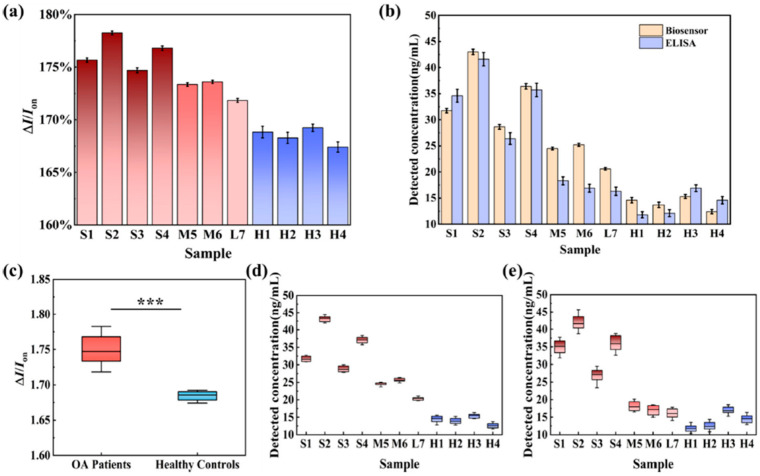
Detection of CRTAC1 protein: (**a**) The biosensor’s response was evaluated using clinical serum samples from patients with severe (S1–S4), moderate (M5–M6), and mild (L7) osteoarthritis (OA), as well as from healthy individuals (H1–H4), with each group comprising 10 replicates (n = 10). (**b**) A comparative analysis was conducted between CRTAC1 concentrations measured by the biosensor and those determined via ELISA. (**c**) A statistically significant difference in biosensor signal intensity was observed between OA patients and healthy controls (n = 10), with *p* < 0.001. Results are expressed as mean ± standard deviation (SD). (**d**,**e**) Quantitative detection of CRTAC1 levels in serum samples using the biosensor (**d**) and ELISA (**e**). Box plots represent interquartile ranges (25th to 75th percentiles), with whiskers indicating the 10th to 90th percentiles; medians are denoted by horizontal lines within the boxes (reproduced with permission from [55]).

Lv et al. [55] recognized osteoarthritis as a prevalent degenerative joint disorder with substantial impact on joint functionality and patient quality of life, in which Cartilage acidic protein 1 (CRTAC1) emerged as a promising biomarker that was correlated with disease progression and surgical outcomes. The research team developed an innovative SWCNT-FET-based biosensing platform for label-free CRTAC1 detection, addressing the limitations of conventional diagnostic approaches in sensitivity and specificity. Antibody-functionalized semiconducting nanotube films served as the transduction element, exhibiting exceptional electrical characteristics and molecular recognition capabilities. A systematic evaluation demonstrated the biosensor’s superior analytical performance compared to standard immunoassay techniques, including an enhanced detection sensitivity, improved measurement accuracy, and reduced analytical variability. Clinical validation studies confirmed the platform’s capability to reliably differentiate osteoarthritis patients from healthy controls through direct serum analysis (Figure 8). The investigation further established the biosensor’s potential for multiplexed biomarker detection, suggesting broader applicability in clinical diagnostics beyond osteoarthritis monitoring. These findings position CNT-FET biosensors as a transformative approach for degenerative joint disease management, offering significant advantages over existing diagnostic methodologies in both performance characteristics and practical implementation.

### 4.4. Cardiovascular and Metabolic Disorder Detection

Cardiovascular diseases and metabolic disorders require continuous monitoring for effective management. Traditional diagnostic methods can be slow and resource-intensive. CNT-FET biosensors provide the real-time, high-sensitivity detection of cholesterol for heart disease and calcium levels for hypocalcemia. Their integration into wearable devices enhances early detection and personalized healthcare.

**Figure 9:**
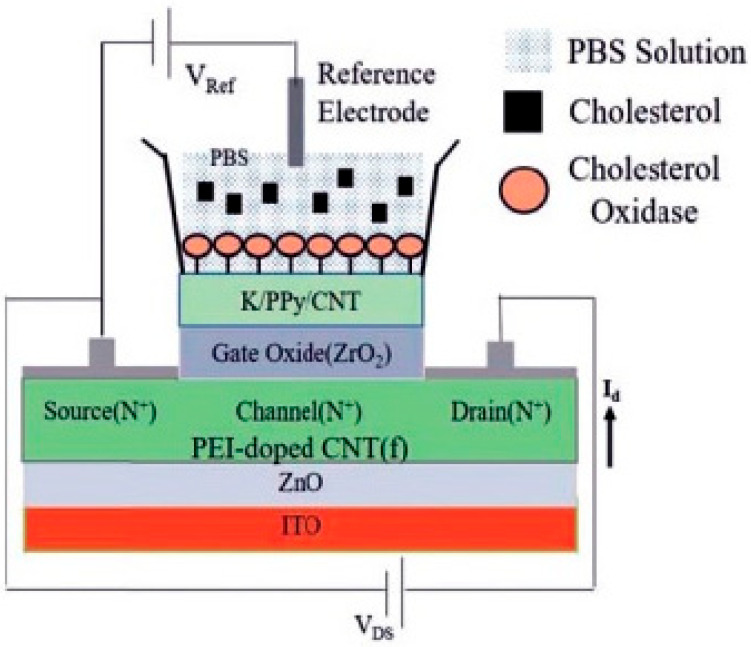
Schematic diagram of enzyme-modified FET for cholesterol detection (reproduced with permission from [49]).

Keshwani et al. [49] developed an innovative ion-sensitive FET biosensor for cholesterol detection utilizing CNTs with high-κ dielectric properties. They fabricated the device through solution-based fabrication methods, incorporating polyethylenimine-doped CNTs as the conductive channel and a nanocomposite membrane consisting of potassium-doped nanotubes with polypyrrole (Figure 9). Following the immobilization of cholesterol oxidase enzymes in phosphate-buffered saline solution, a comprehensive electrical characterization revealed the biosensor’s exceptional performance characteristics. The platform demonstrated broad linear detection range capabilities coupled with significant sensitivity parameters and rapid-response kinetics. A systematic evaluation confirmed the device’s long-term operational stability and excellent molecular discrimination properties, as evidenced by minimal interference from competing analytes. These findings established the CNT-FET architecture as a promising analytical platform for cholesterol monitoring applications, combining the advantages of nanomaterial-enhanced sensitivity with robust biosensing performance.

Wang et al. [6] identified hypocalcemia as a critical metabolic disorder in postpartum dairy cattle resulting from abrupt decreases in blood calcium levels, potentially leading to severe health complications. They addressed the need for cost-effective and user-friendly diagnostic tools by developing a novel microfluidic FET biosensor incorporating semiconducting SWCNTs and calcium-specific DNAzymes. To overcome the inherent limitations of cellulose paper substrates in aqueous environments, the investigators employed an octadecyltrichlorosilane treatment to create a superhydrophobic surface with enhanced mechanical durability. The biosensor platform utilized the pyrene carboxylic acid functionalization of CNTs through π-π stacking interactions, providing anchoring sites for DNAzyme immobilization via carboxyl–amine conjugation. Two distinct DNAzyme configurations with varying calcium sensitivities were integrated into a dual-sensor array, significantly improving measurement reliability by compensating for matrix interference effects in bovine blood samples. The systematic optimization of detection parameters yielded a biosensor capable of quantifying physiologically relevant calcium concentrations with a high degree of specificity. Clinical validation demonstrated the platform’s effectiveness in monitoring calcium dynamics in bovine blood, establishing its potential as a diagnostic tool for postpartum hypocalcemia management in dairy cattle.

**Figure 10:**
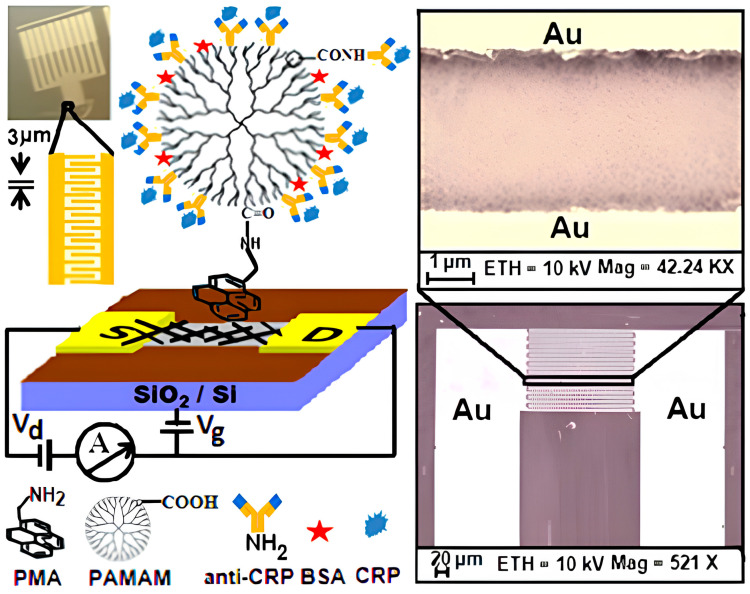
A schematic representation of the PAMAM-functionalized SWCNT-FET biosensor is presented, accompanied by an SEM image depicting the SWCNT network bridging gold source and drain electrodes spaced 3 μm apart (reproduced with permission from [56]).

Rajesh et al. [56] reported the fabrication of an SWCNT-based FET that was surface-functionalized using a polyamidoamine (PAMAM) dendrimer featuring 128 terminal carboxyl groups, which served as anchoring sites for the site-specific immobilization of anti-C-reactive protein (anti-CRP) antibodies (Figure 10). Upon exposure to varying concentrations of C-reactive protein (CRP), a clinically relevant, concentration-dependent reduction in the drain current was recorded, with the sensor exhibiting a limit of detection around 85 pM. The system demonstrated a sensitivity of approximately a 20% change in normalized current (ΔI/I) per logarithmic unit of CRP concentration, consistent with the localized gating of the SWCNT channel induced by the antigen–antibody interaction. Furthermore, the calculated dissociation constant (K_d_ = 0.31 ± 0.13 μg/mL) indicated a strong binding affinity between the FET-bound antibodies and CRP, attributed to the high-density and spatially favorable orientation of capture probes enabled by the 3D PAMAM dendritic scaffold.

The prior discussion of CNT-FET-based biosensing platforms is summarized in Table 1.

## 5. Challenges and Future Perspectives

Despite the remarkable progress in CNT-FET biosensors, several challenges remain that must be addressed to enable their widespread adoption in clinical diagnostics and wearable biosensing applications. These challenges include material synthesis and processing limitations, device stability, reproducibility, biocompatibility, and large-scale manufacturing constraints. Addressing these hurdles is critical for advancing CNT-FET biosensors from laboratory prototypes to commercially viable diagnostic tools.

One of the primary challenges in CNT-FET biosensor development is the difficulty of producing high-purity [57], well-aligned semiconducting CNTs with consistent electronic properties [58]. Despite significant progress in CNT synthesis, challenges remain in achieving uniform chirality control [59], large-scale production [60], and reproducibility for commercial applications. CNT synthesis methods, such as CVD, often yield a mixture of metallic and semiconducting nanotubes [61], leading to device variability and inconsistent sensor performance. Although purification techniques, such as polymer-assisted sorting [62] and density gradient centrifugation [63], have improved the semiconducting CNT yield, these methods remain costly and time-consuming. Future research should focus on scalable, cost-effective strategies such as employing modified cotton [64] to produce highly purified semiconducting CNTs with uniform electronic characteristics, ensuring reproducibility in biosensor fabrication.

Another significant challenge is the long-term stability and reliability of CNT-FET biosensors in real-world applications. The functionalization of CNTs with bio-recognition elements is essential for target specificity, but these functionalized surfaces can degrade over time [65], leading to sensor performance deterioration. Factors such as non-specific adsorption, biofouling, and environmental variability further impact device stability. Developing antifouling surface coatings and optimizing immobilization techniques to enhance the durability of bio-functionalized CNTs is crucial for improving sensor longevity and reliability [66]. Additionally, integration with microfluidic systems and protective encapsulation strategies may help maintain a stable performance in complex biological samples [67].

Reproducibility and batch-to-batch variation remain major obstacles in CNT-FET biosensor manufacturing [68]. Differences in CNT deposition techniques, variations in functionalization protocols, and inconsistencies in fabrication processes can lead to significant deviations in sensor response. Standardized fabrication techniques and advanced nanomanufacturing methods, such as roll-to-roll printing [69] and self-assembly approaches, are needed to improve reproducibility and scalability. Furthermore, the development of automated quality control measures for device screening and calibration will enhance the reliability of CNT-FET biosensors in commercial applications.

Biocompatibility is a critical consideration for in vivo and wearable biosensing applications, in which CNTs, despite their exceptional electrical and mechanical properties, raise concerns regarding long-term toxicity and immunogenicity [70]. Recent studies have indicated that CNT toxicity is influenced by factors such as size, aspect ratio, surface charge, aggregation state, and residual catalytic impurities [71]. Long-term in vivo experiments have reported issues such as inflammation, oxidative stress, and organ accumulation, particularly in the lungs, liver, and spleen [72]. To mitigate these effects, various surface modification strategies have been explored. Functionalization with biocompatible polymers such as polyethylene glycol (PEG) [73], chitosan [74], or poly(lactic-co-glycolic acid) (PLGA) [75] can enhance dispersion, reduce immune responses, and improve overall biocompatibility. Lipid coatings [76] and conjugation with biomolecules like peptides [77] further reduce cytotoxicity while maintaining sensor performance. Additionally, hybrid nanostructures combining CNTs with materials like gold nanoparticles have shown improved safety profiles [78]. Some studies have even explored enzyme-mediated degradation as a strategy for biodegradability [79]. Nonetheless, comprehensive long-term studies are still needed to fully understand the biological fate of CNTs and ensure their safe application in healthcare monitoring systems [80].

The scalability and commercialization of CNT-FET biosensors require overcoming challenges related to cost-effective mass production and integration with existing diagnostic platforms [81]. Current fabrication methods are often labor-intensive and require specialized equipment, limiting their feasibility for large-scale deployment. One promising strategy to tackle these issues is the implementation of roll-to-roll (R2R) manufacturing techniques [82]. R2R processing allows for the continuous production of materials and devices on flexible substrates, which significantly enhances throughput and reduces production costs [83]. This method is particularly suitable for large-area applications such as flexible electronics [84], sensors, and other devices [85]. In addition to R2R manufacturing, inkjet printing [86] and screen printing technologies [87] offer scalable and material-efficient fabrication routes. These additive manufacturing techniques minimize waste and are compatible with various substrates, including plastics and textiles, supporting low-cost, high-volume production. The development of flexible and stretchable CNT-FET biosensors for wearable applications [88] further requires innovative manufacturing approaches, such as inkjet printing and flexible electronics fabrication. Collaborative efforts between researchers, industry partners, and regulatory bodies are essential to streamline commercialization pathways, ensuring that CNT-FET biosensors meet regulatory standards and market demands.

The potential of CNT detection technology in large-scale applications is significant, driven by advances in synthesis and manufacturing. Recent developments in scalable production methods, such as R2R processing, inkjet printing, and screen-printing technologies, have reduced costs and improved the uniformity of CNT films, paving the way for industrial adoption. However, challenges remain, including the need for the precise control over CNT chirality and alignment to ensure consistent sensor performance. Integration into existing technological frameworks, such as Internet of Things (IoT) platforms, could enable real-time monitoring in environmental, healthcare, and industrial settings [89]. Despite these opportunities, regulatory hurdles and potential toxicity concerns must be addressed to ensure safe deployment.

Future perspectives in CNT-FET biosensor technology are increasingly shaped by the integration of artificial intelligence (AI) [90], machine learning (ML) [91], and IoT [92] frameworks, which together enhance sensing accuracy, efficiency, and real-time usability. Recent advancements in AI-driven data processing have enabled the development of sophisticated algorithms capable of handling large volumes of complex, multidimensional biosensor data [93]. These algorithms, including deep learning models and support vector machines, can effectively filter noise, detect subtle biomarker patterns, and improve signal interpretation, thereby significantly increasing diagnostic accuracy and reliability. In parallel, the fusion of CNT-FET biosensors with wireless communication modules (e.g., Bluetooth, Wi-Fi, and 5G) has led to the emergence of IoT-enabled healthcare monitoring systems [94]. These systems support the real-time, continuous monitoring of physiological parameters and the seamless transmission of health data to cloud-based platforms for remote analysis and feedback [95]. This integration paves the way for intelligent, connected diagnostic tools capable of early disease detection, personalized treatment planning, and improved patient outcomes. Furthermore, these advancements align with the broader vision of smart healthcare ecosystems, enabling proactive and preventive care through seamless human–device–data interactions.

Another exciting direction for CNT-FET biosensors is multi-analyte detection and multiplexed sensing platforms [96]. Current biosensor designs often focus on detecting a single biomarker; however, many diseases involve complex biomolecular signatures that require the simultaneous detection of multiple analytes. Engineering CNT-FET biosensors with multiple functionalization sites and integrating them with lab-on-a-chip systems [97] can enable comprehensive biomarker profiling, enhancing diagnostic precision and disease characterization.

Compared with other detection technologies, CNT-based sensors offer notable cost optimization advantages due to their unique material properties and fabrication methods. CNT sensors can be produced using scalable [98], low-cost techniques [99] which avoid expensive cleanroom requirements and complex lithographies, enabling large-area manufacturing suitable for mass production. Their nanoscale size and high sensitivity allow for minimal material usage and low power consumption [100], reducing both production and operational costs. Furthermore, CNTs operate efficiently at room temperature, eliminating the need for costly heating elements, which is common in some traditional sensors. The compatibility of CNTs with existing microfabrication and electronic integration processes facilitates their seamless incorporation into current device architectures, enhancing manufacturing efficiency and lowering overall expenses [101]. Collectively, these factors position CNT-based detection technology as a cost-effective alternative to conventional sensors, supporting its potential for widespread, large-scale deployment across diverse applications such as environmental monitoring, healthcare diagnostics, and industrial safety.

## 6. Conclusions

In conclusion, while CNT-FET biosensors have demonstrated remarkable potential in diverse biosensing applications, addressing challenges related to material synthesis, stability, reproducibility, biocompatibility, and scalability is essential for their practical implementation. Continued interdisciplinary research and collaboration will drive innovations in CNT-based biosensing technologies, paving the way for their integration into next-generation diagnostic and healthcare solutions. The future of CNT-FET biosensors lies in their ability to provide the rapid, sensitive, and cost-effective detection of biomolecules, ultimately transforming the landscape of personalized medicine and point-of-care diagnostics.

## Figures and Tables

**Table 1:** Key parameters of CNT-FET-based biosensing platforms.

Substrate	Source/Drain Material	Gate Type	Active Layer/ Transducer	Receptor	Target	Sample/Sample Mixed in	LOD	Sensitivity	Detection Range	Response Time	Identified Diseases	Ref
SiO_2_/Si	Ti/Pd/Au	Floating gate	CNT	DNA probe	miRNA21	Plasma	0.87 aM	NA	10 aM to 100 nM	NA	Breast cancer	[48]
SiO_2_/Si	Au	Solution gate	Poly-lysine functionalised CNT	Sulfhydryl aptamer probe	exosomal protein (MUC1)	Plasma	0.34 fg/mL	NA	1 fg/mL to 100 pg/mL	<1 h	Breast cancer	[51]
SiO_2_/Si	Ti/Pd/Au	Liquid gate	CNT	DNA, PMO, PNA probes	BRCA1	Serum	1.38 aM	NA	1 aM to 100 pM	30 min	Breast cancer	[52]
SiO_2_/Si	Ti/Pd/Au	Floating gate	CNT	Monoclonal carcinoembryonic antibody	Carcinoembryonic antigen (CEA)	Serum	72 ag/mL	NA	1 fg/mL to 1 ng/mL	1 h	Lung cancer	[5]
SiO_2_/Si	Ti/Au	Back gated	CNT	SARS-CoV-2 S1 antibody	SARS-CoV-2 S1 antigen	Saliva	4.12 fg/mL	NA	0.1 fg/mL to 5.0 pg/mL	2 to 3 min	COVID-19	[47]
SiO_2_/Si	Au	Liquid gate	cGQD coupled CNT	Polymyxin B	Bacterial endotoxin	Serum	30.3 fg/mL	NA	100 fg/mL to 1 ng/mL	3 min	Fatal fever	[7]
SiO_2_/Si	Ti/Pd/Au	Liquid gate	CNT	Nucleic acid aptamers	Salmonella enterica and Staphylococcus aureus	Pork, Chicken, Beef, Milk, Fish, and Eggs	1 CFU (*S. enterica*) and 1.2 CFUs (*S. aureus*)	NA	10^2^ to 1.28 × 10^4^ CFU/mL	200 s	Foodborne diseases	[46]
SiO_2_/Si	Ti/Au	Back-gate	CNT	Antibodies specific to domoic acid	Domoic acid (Neurotoxin)	Seawater	10 ng/L	NA	10 to 500 ng/L	15 min	Amnesic shellfish poisoning	[53]
SiO_2_/Si	Ti/Pd/Au	Liquid gate	CNT	Oligonucleotide aptamers	AD core blood biomarkers of β-amyloid (Aβ)	Serum	50 aM	NA	1 fM to 10 pM	30 min	Alzheimer’s disease	[54]
SiO_2_/Si	Ti/Pd/Au	Bottom-gated	SWCNT	CRTAC1 antibody	Cartilage Acidic Protein 1 (CRTAC1)	Serum	0.2 fg/mL	NA	1 fg/mL to 100 ng/mL	30 min	Osteoarthritis	[55]
ITO	Al	Solution-gated	Polyethylenimine (PEI) doped CNT	Potassium doped CNT with polypyrrole	Cholesterol	PBS	0.23 mM	60 mV/decade	0.5 to 25 mM	~1 s	Heart disease	[49]
Cellulose paper	Ag	Back gated	SWCNT	Dual-MFB	Ca^2+^	Cow blood	10.7 μM	NA	25 μM to 5 mM	7 min	Hypocalcemia	[6]
SiO_2_/Si	Ti/Au	Back gated	SWCNT	anti-CRP	CRP	CRP in Phosphate Buffer Saline	85 pM	20% change in current per decade	0.01 to 10 µg/mL	10 min	Inflammatory diseases	[56]

## Data Availability

Not applicable.
